# Spike-Timing Precision and Neuronal Synchrony Are Enhanced by an Interaction between Synaptic Inhibition and Membrane Oscillations in the Amygdala

**DOI:** 10.1371/journal.pone.0035320

**Published:** 2012-04-26

**Authors:** Steven J. Ryan, David E. Ehrlich, Aaron M. Jasnow, Shabrine Daftary, Teresa E. Madsen, Donald G. Rainnie

**Affiliations:** Division of Behavioral Neuroscience and Psychiatric Disorders, Department of Psychiatry and Behavioral Sciences, Yerkes Research Center, Emory University School of Medicine, Atlanta, Georgia, United States of America; Consejo Superior de Investigaciones Cientificas - Instituto Cajal, Spain

## Abstract

The basolateral complex of the amygdala (BLA) is a critical component of the neural circuit regulating fear learning. During fear learning and recall, the amygdala and other brain regions, including the hippocampus and prefrontal cortex, exhibit phase-locked oscillations in the high delta/low theta frequency band (∼2–6 Hz) that have been shown to contribute to the learning process. Network oscillations are commonly generated by inhibitory synaptic input that coordinates action potentials in groups of neurons. In the rat BLA, principal neurons spontaneously receive synchronized, inhibitory input in the form of compound, rhythmic, inhibitory postsynaptic potentials (IPSPs), likely originating from burst-firing parvalbumin interneurons. Here we investigated the role of compound IPSPs in the rat and rhesus macaque BLA in regulating action potential synchrony and spike-timing precision. Furthermore, because principal neurons exhibit intrinsic oscillatory properties and resonance between 4 and 5 Hz, in the same frequency band observed during fear, we investigated whether compound IPSPs and intrinsic oscillations interact to promote rhythmic activity in the BLA at this frequency. Using whole-cell patch clamp in brain slices, we demonstrate that compound IPSPs, which occur spontaneously and are synchronized across principal neurons in both the rat and primate BLA, significantly improve spike-timing precision in BLA principal neurons for a window of ∼300 ms following each IPSP. We also show that compound IPSPs coordinate the firing of pairs of BLA principal neurons, and significantly improve spike synchrony for a window of ∼130 ms. Compound IPSPs enhance a 5 Hz calcium-dependent membrane potential oscillation (MPO) in these neurons, likely contributing to the improvement in spike-timing precision and synchronization of spiking. Activation of the cAMP-PKA signaling cascade enhanced the MPO, and inhibition of this cascade blocked the MPO. We discuss these results in the context of spike-timing dependent plasticity and modulation by neurotransmitters important for fear learning, such as dopamine.

## Introduction

The basolateral complex of the amygdala (BLA) is a critical part of the neural circuit regulating fear learning [1]–[5], and recent evidence suggests that oscillatory activity of neurons in this region plays a key role in regulating affect in awake, behaving animals (for review, see [6]). More specifically, it is now evident that the amygdala, hippocampus, and prefrontal cortex produce coordinated high delta/low theta (4–5 Hz) oscillations during acquisition [7] and retrieval [8] of learned fear, which then diminish over the course of subsequent extinction learning. Significantly, phase-locked theta stimulation applied simultaneously to the amygdala and hippocampus disrupts fear extinction and prolongs the expression of learned fear [9], further supporting a role of synchronized neural activity in the processes of fear learning and extinction. Moreover, synchronous theta oscillations during REM sleep in the period between fear acquisition and retrieval correlate with changes in fear expression, suggesting that theta oscillations are critical for successful consolidation of fear memory [10]. Despite the importance of these low frequency oscillations to amygdala function and emotional learning, the mechanisms by which the BLA circuit generates rhythmic activity are largely unknown.

A common mechanism for generating network oscillations utilizes coordinated inhibitory input across multiple neurons to synchronize their action potential firing [11]–[17]. The BLA is organized to exploit this phenomenon through the rhythmic interaction of excitatory principal neurons and inhibitory interneurons. BLA principal neurons exhibit compound, rhythmic, inhibitory postsynaptic potentials (IPSPs) that occur at a baseline frequency of 0.5–4 Hz that is sensitive to modulation by dopamine and serotonin [18]–[20]. These rhythmic IPSPs are driven by action potentials in local, burst-firing interneurons, which we have previously shown to express parvalbumin (PV^+^) [21]. PV^+^ interneurons have several characteristics that enable them to influence the activity of large networks of BLA principal neurons synchronously: first, these interneurons make up approximately 40% of the total interneuron population and are distributed throughout the BLA; second, each PV^+^ interneuron can innervate the soma and axon hillock of approximately 150 principal neurons [22]; finally, these interneurons are coupled electrically by gap junctions to create a functional syncytium [23]–[25]. Significantly, we and others have shown that, in paired recordings of rat BLA principal neurons, spontaneous IPSPs are highly synchronized [26], [27], suggesting that the output of PV^+^ interneurons may coordinate the activity of large numbers of principal neurons.

Synchronous IPSPs in large groups of BLA principal neurons could also facilitate network oscillations by interacting with intrinsic oscillations in principal neurons to promote rhythmic firing. Intrinsic membrane potential oscillations (MPOs) have been shown to improve spike-timing precision [28], which is, in turn, important for spike-timing dependent plasticity [29] and signal processing in neural networks [30]. BLA principal neurons display a highly consistent MPO [31], [32] and an intrinsic resonance [33], both in the same high delta/low theta frequency band as network oscillations observed during fear learning. If these MPOs were to occur synchronously in groups of BLA neurons, network activity should be promoted at this highly relevant frequency. Considering that groups of cells can have their firing activity entrained by synchronized IPSPs [34], we chose to investigate the possibility that synchronized, rhythmic IPSPs entrain and phase-lock MPOs and coordinate firing activity in BLA principal neurons. Furthermore, we examine the underlying currents and intracellular signaling cascades regulating these phenomena and discuss potential links to synaptic plasticity and fear learning.

## Materials and Methods

### Animals and housing conditions

Whole cell patch clamp recordings were obtained from 76 neurons from 48 rodents, and 46 neurons from 13 primates. Rodent experiments were conducted on tissue from male Sprague-Dawley rats at 5–7 weeks of age. All rats were group-housed 4 per cage in Plexiglas cages with corn cob (Bed-O-Cob) bedding. Rats had access to food and water *ad libitum*, and were maintained in a temperature controlled colony room on a 12∶12 light∶dark cycle. The primate tissue for this study was obtained from juvenile (18–36 months) *Macaca mulatta* monkeys of both genders. Primates used in this study were born into the breeding colony housed at the Yerkes National Primate Research Center Field Station and raised in normal social groups. They were provided with *ad libitum* access to food and water and monitored by the Yerkes Veterinary Staff. Animals used in this study were selected for sacrifice by the veterinary staff for failure to thrive and/or chronic diarrhea refractory to treatment as part of the animal care end-points approved for our monkey colony. Once identified, the animals were moved to the Yerkes Main Station and scheduled for sacrifice within the week.

**Figure 1:**
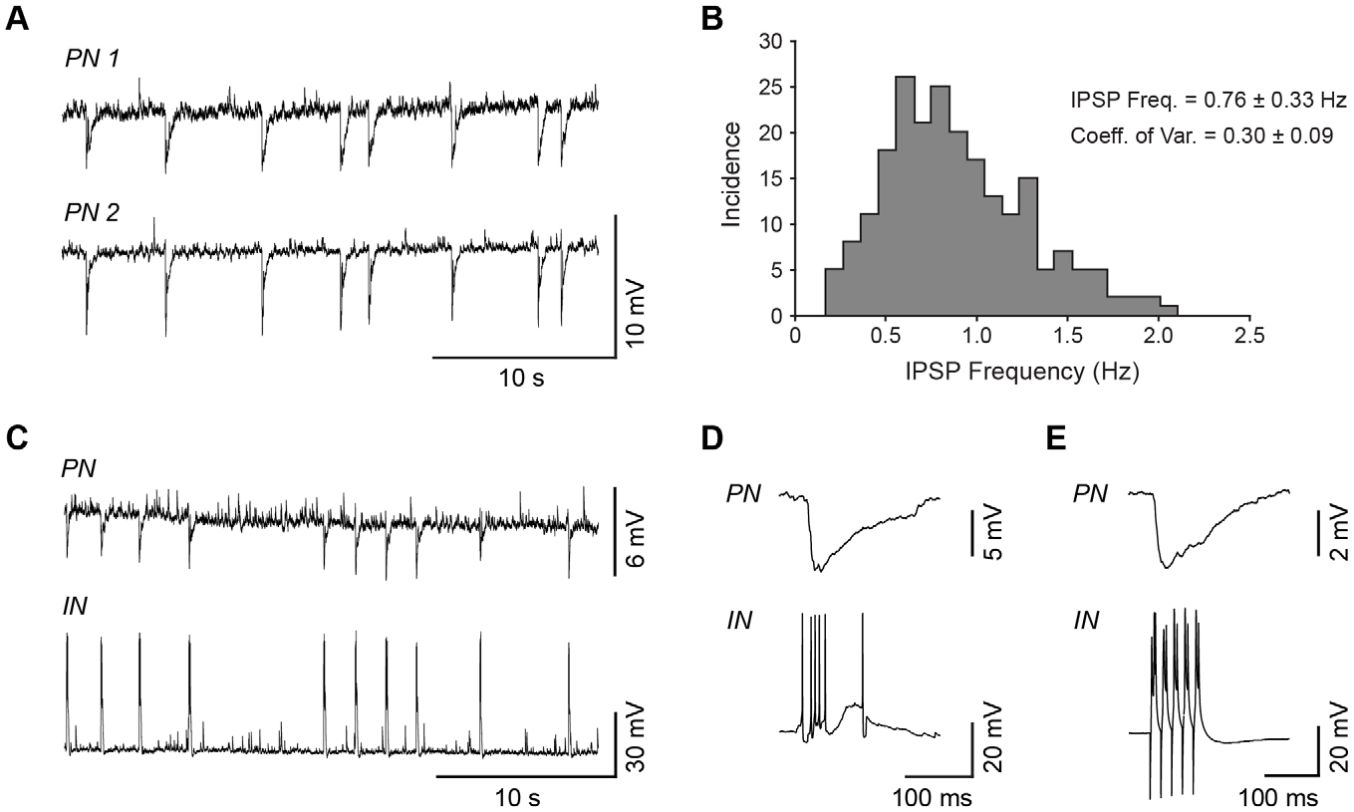
Spontaneous, compound IPSPs in the BLA were synchronized across principal neurons and with bursts in inhibitory interneurons. (A) A representative pair of primate BLA principal neurons, held at −60 mV, showing compound IPSPs that are rhythmic and highly synchronized, observed during gap-free recordings. (B) A histogram plotting instantaneous frequency of compound IPSPs during 30-second recordings from 12 primate BLA principal neurons. (C) Paired recordings in the primate BLA of a principal neuron receiving compound IPSPs and a burst-firing parvalbumin interneuron, both held at −60 mV. (D) An example of a burst-IPSP pair shown at higher temporal resolution. (E) A compound IPSP can be induced in a BLA principal neuron by using current injection to drive bursting activity in the interneuron.

**Figure 2:**
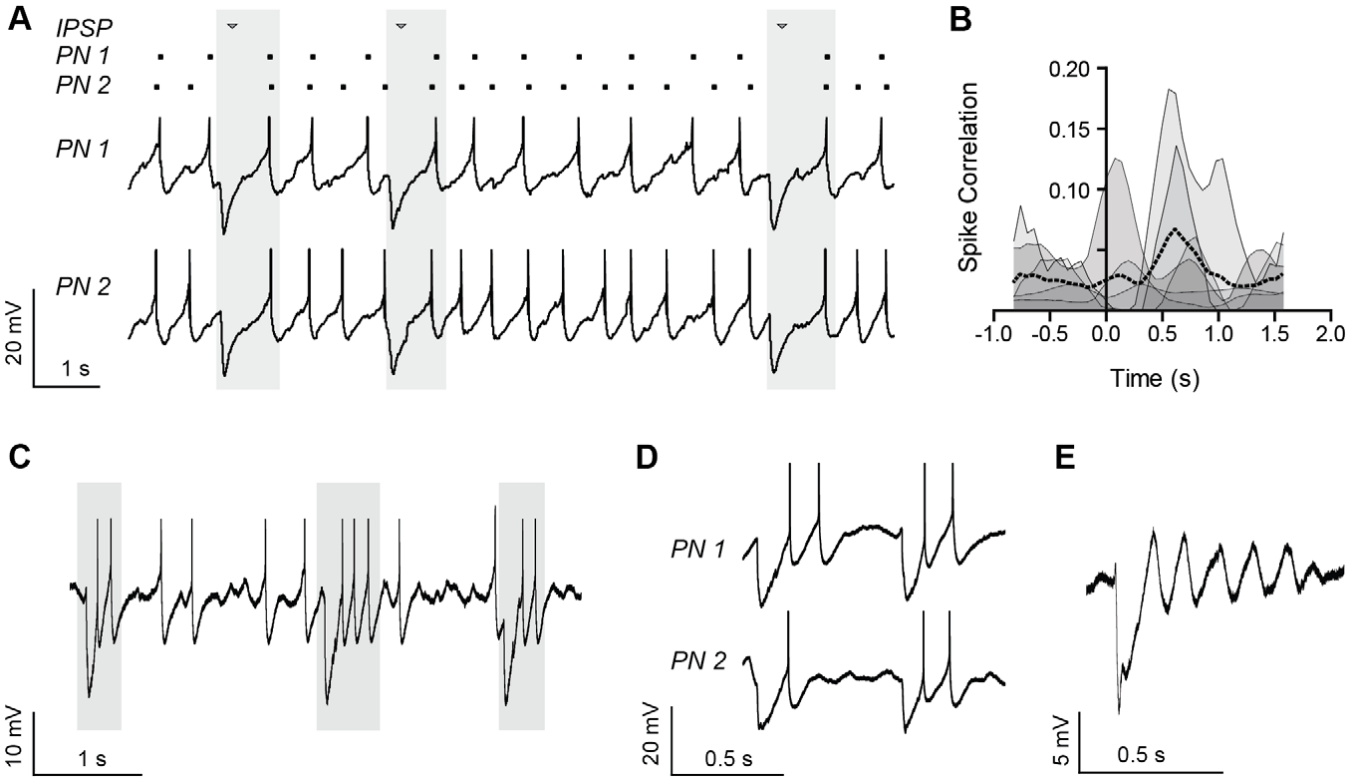
Spontaneous, compound IPSPs coordinated spike timing and promoted rhythmic firing in the primate BLA. (A) Spontaneous, compound IPSPs exhibited by a representative pair of primate BLA projection neurons, depolarized to action potential threshold (−45 to −40 mV) using a DC current injection. A raster plot highlights the relative synchrony of spikes following the IPSPs, highlighted in gray boxes. Action potentials are cropped at −30 mV (n = 6). (B) A spike correlation metric (see Methods) is plotted for 6 pairs of primate BLA principal neurons exhibiting compound IPSPs and depolarized to threshold, as in *A*. Correlation is plotted for each pair as an individual, smoothed trace (thin black lines) representing the mean correlation surrounding every spontaneous, compound IPSP, with the peak of each IPSP aligned to time 0. The mean of all 6 pairs is superimposed as a dotted black line. (C–D) A representative single (C, n = 4) and pair of (D, n = 2) primate BLA principal neurons exhibiting rhythmic firing upon rebound from spontaneous, compound IPSPs. Neurons were depolarized to threshold, as in *A*. IPSPs and rebound firing are highlighted with gray boxes in *C*. Action potentials were cropped at −30 mV. (E) A primate BLA principal neuron, depolarized as in *A*, exhibiting a damped membrane potential oscillation in response to a spontaneous, compound IPSP.

Experiments for Figures 1 & 2 were performed in both rat and primate tissue, and the remainder of experiments were performed exclusively in rat tissue (see figure legends for details). The care of the animals and all anesthesia and sacrifice procedures in this study were performed according to the National Institutes for Health Guide for the Care and Use of Laboratory Animals and were approved by the Institutional Animal Care and Use Committee of Emory University.

### Electrophysiological procedures

*Preparation of acute BLA slices.* To obtain slices from the rat basolateral amygdala, animals were decapitated under isoflurane anesthesia (Abbott Laboratories, North Chicago, IL). The brains were rapidly removed and placed in ice-cold kynurenic acid-based artificial cerebrospinal fluid (KA-ACSF), which contained (in mM): NaCl (130), KCl (3.5), KH_2_PO4 (1.1), MgCl_2_ (6.0), CaCl_2_ (1.0), NaHCO_3_ (30), glucose (10), thiourea (0.8), sodium pyruvate (2), ascorbic acid (0.4), and kynurenic acid (2). The glutamatergic antagonist kynurenic acid was included in the KA-ACSF to suppress any excitotoxic effects of glutamate release that may occur due to tissue slicing. A block of tissue containing the BLA was then mounted in a Leica VTS-1000 vibrating microtome (Leica Microsystems, Bannockburn, IL), and 350 µm coronal slices were cut. Slices were hemisected and hand-trimmed to remove excess tissue dorsal to the amygdala. For the primate basolateral amygdala, the animals were sacrificed with an overdose of pentobarbital (100 mg/kg) and hand-cut blocks of tissue from the medial temporal lobe were mounted in a vibratome and 400 µm coronal slices were cut as previously described [19]. Slices from both species were transferred to a holding chamber containing KA-ACSF at 32°C and gassed with a 95%/5% O_2_/CO_2_ mixture for 40 min before being placed in oxygenated regular ACSF (ACSF) at room temperature containing (in mM): NaCl (130), KCl (3.5), KH_2_PO_4_ (1.1), MgCl_2_ (1.3), CaCl_2_ (2.5), NaHCO_3_ (30), glucose (10), thiourea (0.8), sodium pyruvate (2), and ascorbic acid (0.4).

*Recording procedures.* For recording, slices were placed in a Warner Series 20 recording chamber (Warner Instruments, Hamden, CT) mounted on the fixed stage of a Leica DM-LFS microscope (Leica Microsystems, Bannockburn, IL). Slices were fully submerged and continuously perfused at a rate of 1–2 mL/min with heated (32°C) and oxygenated ACSF. Neurons were selected for recording under IR-DIC illumination with a 40× water immersion objective. Images were captured with a Hamamatsu Orca ER CCD camera (Hamamatsu, Tokyo, Japan) controlled by SimplePCI software (Compix, Sewickley, PA). Whole cell patch-clamp recordings were conducted using thin-walled borosilicate glass-patch electrodes (WPI, Sarasota, FL) which were pulled on a P-97 Flaming/Brown micropipette puller (Sutter Instruments, Novato, CA). Patch electrodes had resistances ranging from 4–7 MΩ when filled with standard patch solution that contained (in mM): K-gluconate (138), KCl (2), MgCl_2_ (3), phosphocreatine (5), K-ATP (2) NaGTP (0.2), HEPES (10), and biocytin (3 mg/mL). The patch solution was adjusted to a pH of 7.3 with KOH and had a final osmolarity of approximately 280 mOsm. Junction potentials were offset manually prior to patching neurons. Access resistances were monitored throughout recordings and neurons with more than a 15% change were discarded. In the case of paired recordings, two neurons were selected for patching within a single 40× visual field. Neuronal types were pre-selected based on somatic morphology, and type was verified based on electrophysiological profile, as described previously for rat [35] and primate [19].

All recordings were performed in principal neurons of the basolateral nucleus of the amygdala, contained in the basolateral complex. Recordings were obtained using an Axopatch-700A amplifier (Molecular Devices, Sunnyvale, CA), a Digidata 1320A A/D interface, and pClamp 10 software (Molecular Devices). For all experiments, whole cell patch-clamp configuration was established, and cell responses were recorded in either current clamp or voltage clamp mode. Data were filtered at 5 kHz in current clamp and 2 kHz in voltage clamp, and sampled at a rate of 10 kHz. Neurons were excluded from analysis if their resting membrane potential (V_m_) was more positive than −55 mV or if their action potentials did not surpass +5 mV.

*Drug Application.* Drugs were applied by gravity perfusion at the required concentration in the circulating ACSF. Drugs used: cesium chloride (CsCl), 5 mM; nickel chloride (NiCl_2_), 500 µM; 4-aminopyridine (4-AP), 100–500 µM; tetrodotoxin (TTX), 1 µM; tetraethylammonium chloride (TEA-Cl), 20 mM; forskolin, 10 µM; dideoxy-forskolin, 10 µM; 1,2-bis(o-aminophenoxy)ethane-N,N,N′,N′-tetraacetic acid (BAPTA), 5 mM purchased from Sigma–Aldrich (St. Louis, MO); 6,7-dinitroquinoxaline-2,3-dione (DNQX), 20 µM; RS-CPP, 10 µM; CGP 52432, 2 µM; 4-(N-ethyl-N-phenylamino)-1,2-dimethyl-6-(methylamino) pyridinium chloride (ZD7228), 60 µM; (1R,4R,5S,6R)-4-amino-2-oxabicyclo[3.1.0]hexane-4,6-dicarboxylic acid (LY379268), 50 µM; 8-Br-cAMP, 5–10 µM; and (R)-adenosine, cyclic 3′,5′-(hydrogenphosphorothioate) triethylammonium (cAMPs-RP), 25 µM purchased from Tocris (Ellisville, MO). All drugs were stored frozen as concentrated stock solutions in dH_2_O except DNQX, which was made in 50% dimethyl sulfoxide and buffered to pH 7.3.

*Spike-timing precision, resonance, and oscillations.* To assess the effect of IPSPs on spike-timing precision, repetitive action potentials were evoked with a depolarizing, square-wave current step of amplitude set to evoke 4–8 Hz firing from a holding potential of −60 mV. The current injections were repeated five times with an inter-event interval of 10 seconds. To examine the effect of synaptic inhibition on spike-timing precision, the current in the depolarizing step was transiently removed for 15 ms and then ramped back over 100 ms to the command amplitude to mimic compound spontaneous IPSPs observed in BLA principal neurons. Alternatively, pharmacologically isolated, compound, synaptic IPSPs were evoked using electrical stimulation within the dorsal BLA, just medial to the external capsule. The two IPSPs in each sweep were applied 550 and 1415 ms into the depolarizing step, separated by 865 ms start-to-start (∼1.2 Hz), to mimic the frequency of spontaneous IPSPs previously observed in our laboratory.

To examine the membrane potential oscillation of BLA principal neurons, cells were held at −60 mV and injected with the same transient (2.5 s) square-wave depolarizing current pulse as described above. TTX (1 µM) was included in all experiments investigating the membrane oscillation. The voltage response to the DC current pulse was recorded and characterized in regular ACSF and also in varying drug conditions. The amplitude of the current pulse was adjusted such that the steady state membrane potential achieved during current injection was similar before and during drug application (between −40 and −30 mV). Any drug-induced changes in resting membrane potential were compensated for by DC current injection before initiating the transient square-wave depolarizing current pulse to assess the effect on membrane potential oscillations. To assess resonance frequency, principal neurons were held at −60 mV with DC current injection and a sinusoidal frequency sweep of constant current amplitude was injected, increasing from 1–12 Hz over a period of 8 seconds, and the voltage response of the cell was recorded.

#### Data and statistical analysis

The correlation of spontaneous IPSPs and burst-firing from paired recordings of BLA neurons were analyzed by first identifying event times using pClamp software and then using a Pearson product-moment correlation. Spike-timing precision was assessed using a correlation-based metric adapted from Schreiber et al., 2003 [36]. The correlation statistic (R_corr_) was calculated for windows of 200 ms every 66 ms, using the equation (Equation 1) as published. Briefly, spike times from N traces were convolved with a Gaussian filter of pre-determined width (σ) to create spike vectors (*s*). For experiments involving artificial and evoked IPSPs, σ = 6 ms, and to prevent a floor effect due to lower spike rates, for experiments with spontaneous IPSPs, σ = 20 ms. The degree of correlation between the vectors (*s_i_, s_j_*) was calculated using a dot-product normalized to the product of their magnitudes and the number of comparisons being made.
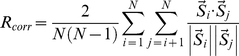
(1)


When calculating spike-timing precision within cells, all 5 traces (N = 5) were compared using this algorithm; when calculating across cells, only the 2 traces (N = 2) which occurred simultaneously were compared, and 5 comparisons were made and averaged. Statistical analyses were performed using a two-way Analysis of Variance (ANOVA), with Bonferroni post-tests to compare across windows and conditions.

Oscillations of the membrane potential of BLA principal neurons were analyzed by means of multi-taper spectral analysis using a custom program that was modified from the Chronux toolbox [37]. The resonance frequency of BLA principal neurons was analyzed with fast Fourier transforms (FFT) in pClamp 10 (Molecular Devices) using a Hamming window. Power spectra (mV^2^/Hz) were converted into standardized Z-scores and peak amplitudes were analyzed using a one-way ANOVA.

## Results

### Primate BLA principal neurons receive spontaneous, synchronized, rhythmic IPSPs that coordinate action potential timing

We have shown previously that approximately 80% of principal neurons in slice preparations of the rat BLA receive spontaneous, compound IPSPs that occur rhythmically at frequencies ranging from 0.5–2 Hz, with a mean of 1.2 Hz, in control ACSF [18]. These compound IPSPs were observed in principal neurons with varying intrinsic properties (mean ± SD: input resistance 85±28 MΩ, action potential threshold −43±3.5 mV, action potential half width 0.8±0.1 ms, data not shown). Here we show that compound IPSPs are also observed in 67% of primate BLA slices with a frequency of 0.76±0.33 Hz, similar to the rat (n = 46, **Figure 1A, B**). As in the rat BLA, compound IPSPs in the primate BLA were highly rhythmic, with a coefficient of variation of instantaneous frequency of 0.30±0.09 (n = 12). Compound IPSPs occur synchronously across multiple neurons in the rat BLA [26], [27], and new analysis reveals they have a near perfect correlation in time across pairs of principal neurons (Pearson product-moment correlation, R^2^ = 0.999; n = 11, data not shown). We extend this observation to show that compound IPSPs are also highly synchronized across pairs of primate neurons (**Figure 1A**; Pearson product-moment correlation, R^2^ = 1.0; n = 5, data not shown), suggesting this is an evolutionarily conserved phenomenon.

Using paired recordings from a burst-firing interneuron and a principal neuron, we extend previous observations in the rat BLA [26], [27] to the primate. Here we show that compound IPSPs observed in BLA principal neurons (**Figure 1 C**, upper trace) coincide with rhythmic bursts of action potentials occurring in burst-firing interneurons (lower trace, n = 2; Pearson product-moment correlation, R^2^ = 0.999, data not shown), which we have previously shown in the rat BLA to express the calcium-binding protein PV^+^ [21]. **Figure 1D** illustrates a typical burst-IPSP pair at higher temporal resolution. Compound IPSPs with a similar waveform can also be observed in principal neurons if an interneuron is driven to fire bursts of action potentials by direct current injection (**Figure 1E**). Previously, we have shown that these compound IPSPs were abolished by application of either the GABA_A_ receptor antagonist, bicuculline, or the AMPA receptor antagonist, CNQX, suggesting glutamatergic input drives burst-firing PV^+^ interneurons to release GABA at multiple sites onto BLA principal neurons [26]. Each parvalbumin interneuron can innervate more than 150 BLA principal neurons [38], further suggesting that spontaneous, compound IPSPs are highly synchronized across a larger population of principal neurons than the pairs we show here.

Elsewhere in the brain, IPSPs have been shown to interact with subthreshold membrane potential oscillations (MPOs) to improve stimulus discrimination and action potential precision in neurons [28], [30]. We were therefore interested in the ability of synchronized, compound IPSPs to coordinate firing activity within networks of BLA principal neurons. As illustrated in **Figure 2**, compound IPSPs are capable of coordinating activity in the BLA, improving the temporal coherence of spontaneous action potentials between pairs of primate BLA principal neurons. When neurons were depolarized to threshold for action potential generation, action potentials occurring upon rebound from an IPSP-induced membrane hyperpolarization were highly coincident across cells (**Figure 2A**, shaded regions). To identify periods with consistent spike-timing across cells, we used a correlation-based metric with a sliding window (see Methods [36]), where a value of 1 indicates identical spike-timing and a value of 0 indicates no timing correlation. Action potentials during a window directly following spontaneous, compound IPSPs had improved temporal coherence across pairs of neurons compared to those preceding IPSPs (**Figure 2B**). Interestingly, upon rebound from compound IPSPs, a subpopulation of primate BLA principal neurons (3/11 cells) exhibited an increased and more consistent firing rate (from 3.6 to 7.4 Hz, coefficient of variation from 0.56 to 0.28) (**Figure 2C**). Moreover, clusters of action potentials showing a consistent firing rate and high coherence following compound IPSPs were also observed in 2/6 paired recordings (**Figure 2D**). In the course of these experiments it was noted that compound IPSPs could elicit a damped oscillation on rebound, suggesting the observed effects on action potential patterning may be due to an interaction with an intrinsic MPO (**Figure 2E**).

Together these observations strongly suggest that compound IPSPs coordinate the firing activity of principal neurons in both the rat and primate BLA, and their prevalence and synchrony further suggest that this coordination extends across large groups of principal neurons. To better assess the interactions of compound IPSPs with intrinsic properties of BLA principal neurons, subsequent experiments examined the effects of IPSPs on spike trains in the absence of synaptic noise. Moreover, given the scarcity of primate tissue, all experiments were performed in the rat.

### Compound IPSPs enhance spike-timing precision in rat BLA principal neurons

**Figure 3:**
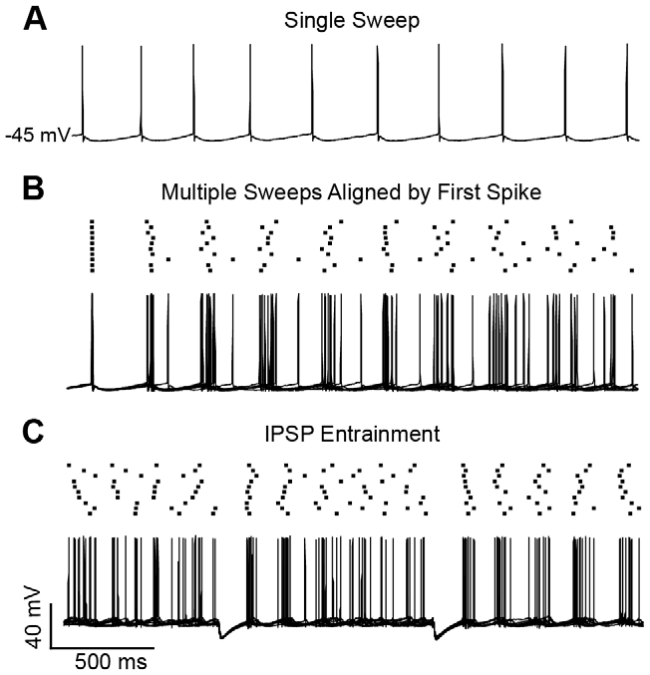
Spike-timing precision diminishes in spike trains and is reset by compound IPSPs. (A) A single sweep recorded from a spiking BLA principal neuron, held at −45 mV by steady-state current injection, displaying a typical regular firing pattern. (B) Multiple sweeps like that in *A* overlaid and aligned by their first spikes. A raster plot illustrates decay of spike-timing reliability. (C) Injection of artificial IPSPs recovers spike-timing precision.

We first examined the effect of IPSPs on the precision of action potential timing in a neuron depolarized to action potential threshold with DC current injection. In order to better isolate the effects of intrinsic currents on spike timing, we blocked synaptic currents with a mixture of glutamate and GABA receptor antagonists (see Methods). As illustrated in **Figure 3A**, BLA principal neurons displayed a regular action potential firing pattern when held at −45 mV. When ten sweeps from the same neuron were aligned using an action potential as the trigger (**Figure 3B**), it was apparent that subtle variations in inter-spike interval accumulated over the course of the train, such that the timing of spikes at the end of the train was less consistent than at the beginning. Conversely, when two simulated IPSPs were injected during 10 sweeps captured randomly in time (**Figure 3C**), the phase of spiking was reset and spike times became much more consistent across sweeps.

**Figure 4:**
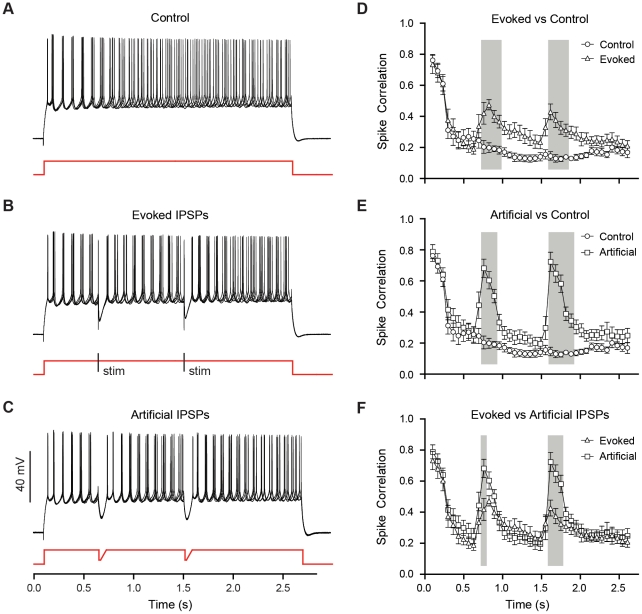
Artificial and evoked compound IPSPs improved spike-timing precision in individual BLA principal neurons. (A) Five superimposed traces from a representative principal neuron, held at −60 mV, showing a train of action potentials in response to a depolarizing current step in the presence of DNQX (20 µM), RS-CPP (10 µM) and CGP (2 µM); note the loss of spike-timing precision as the spike train progresses. (B, C) Similar traces to *A* with the injection of evoked (B) or artificial (C) compound IPSPs to demonstrate improvement of spike-timing precision following a compound IPSP. (D, E, F) Comparisons of spike-timing precision for neurons with no IPSPs (Control, n = 11), evoked IPSPs (n = 11), and artificial IPSPs (n = 11), assessed with a spike correlation metric (see Methods) and plotted as mean ± SEM. Comparisons were made using a two-way ANOVA (see Results), and windows of significant differences (p<0.05) in spike correlation are denoted with grey boxes.

Having established that artificial IPSPs can improve spike-timing precision in free-firing neurons, we next sought to quantify this effect. Specifically, we used transient (2.5 s) steps of injected current to elicit a spike train and determine the effect of IPSPs on spike-timing precision in individual principal neurons, and between pairs of principal neurons. Similar to when neurons are free-firing, the timing of the first few spikes in a train was extremely consistent across sweeps, but the timing of subsequent spikes became less consistent as the train progressed because small variations in the inter-spike interval accumulated (**Figure 4A**). Here we used the same correlation-based metric as described for **Figure 2B**, adapted to compare across five sweeps recorded in a single neuron (see Methods). This analysis revealed that, at the onset of the train, spike-timing was extremely precise with an initial correlation value of 0.75±0.13 (mean ± SD), which then diminished to 0.26±0.20 within 300 ms (**Figure 4D**, Control, n = 11).

We next evaluated spike-timing precision in the presence of stimulus-evoked IPSPs (**Figure 4B**). Electrical stimulation of the dorsolateral BLA in the presence of glutamate receptor antagonists elicited a monosynaptic IPSP in principal neurons that had a similar amplitude and duration to the spontaneous compound IPSPs. We also examined the effects of artificial IPSPs, elicited with hyperpolarizing current injection, on spike-timing precision (**Figure 4C**). Activation of either evoked or artificial IPSPs during the action potential train resulted in a significant improvement in spike-timing precision compared to the control condition (Two-way ANOVA with repeated measures, effect of group: F_2,800_ = 136.3, p<0.0001). Both types of IPSPs significantly increased correlation values relative to the control condition for approximately 270 ms following each IPSP (effect of interaction: F_78,800_ = 4.72, p<0.0001, Bonferroni post-tests). Evoked IPSPs improved correlation values from a baseline of 0.19±0.11 to a peak of 0.47±0.11 (n = 11) immediately following the IPSPs (**Figure 4D**). As illustrated in **Figure 4F**, artificial IPSPs had a more pronounced effect on spike-timing precision than evoked IPSPs, with a peak correlation value of approximately 0.71±0.21 (n = 11) following each IPSP. Only at the peak points of the correlation, however, was there any significant difference in how the two IPSP manipulations affected spike-timing precision.

### Compound IPSPs synchronize the firing activity of multiple BLA principal neurons

**Figure 5:**
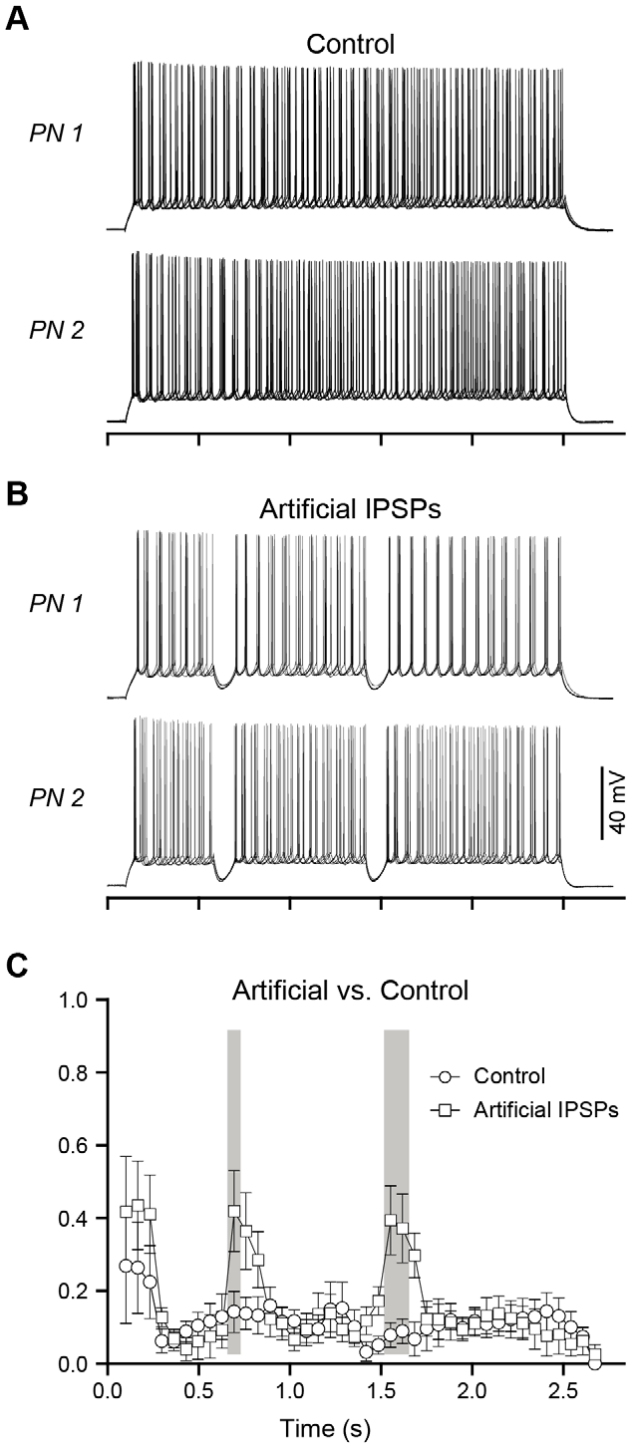
Artificial, compound IPSPs coordinated spike timing across pairs of BLA principal neurons. (A) Five overlaid, consecutive traces of action potentials during paired recordings of BLA principal neurons, held at −60 mV, in response to a depolarizing current injection without IPSPs and (B) with two IPSPs. (C) Spike correlation metric calculated across pairs of neurons when artificial IPSPs are injected compared to the control condition (n = 6 pairs), plotted against time. Comparisons were made using a two-way ANOVA (see Results), and grey boxes denote windows of significant differences (p<0.05) in spike correlation.

We next quantified the ability of compound IPSPs to improve firing coherence across multiple BLA principal neurons, using a similar metric as above to measure the correlation of spike times in simultaneously recorded sweeps across the two neurons. In the absence of IPSPs, spike-timing across BLA neurons showed low coherence, such that the correlation-based metric reached an initial peak of only 0.27±0.39 which then declined rapidly to 0.09±0.07 (n = 6) within 300 ms (**Figure 5C**). The introduction of 2 evoked IPSPs was not able to significantly improve the coherence of spike times between neurons, likely due to the observed inconsistency in the amplitude and duration of the evoked IPSP waveform between neurons (data not shown). Because artificial IPSPs have a highly consistent waveform across pairs of neurons and therefore mimic the consistency of spontaneous, compound IPSPs better than do evoked IPSPs, we also tested the effect of 2 artificial IPSPs on spike-timing. Artificial IPSPs significantly increased the coherence of spike times between pairs of neurons in the period immediately following the IPSPs (Two-way ANOVA with repeated measures, effect of interaction: F_39,200_ = 2.123, p<0.001, Bonferroni post-tests), with an improvement from a baseline of 0.09±0.12 to a peak of approximately 0.42±0.27 (n = 6) in the correlation-based metric (**Figure 5B, D**). These data strongly suggest that synchronized IPSPs enhance spike-timing precision of BLA principal neurons and can serve to entrain the firing activity of multiple neurons, despite inherent differences in their intrinsic electrophysiological properties (e.g., membrane input resistance, time constants of membrane charging, and firing frequency). Based on our prior observation that spontaneous, compound IPSPs not only entrain action potential firing, but also promote rhythmic firing and unmask a damped membrane potential oscillation, we hypothesized that the ability of compound IPSPs to coordinate firing would be facilitated by an interaction with intrinsic oscillatory properties of principal neurons. Therefore, we next characterized the interaction of compound IPSPs with intrinsic oscillatory properties of BLA principal neurons.

### Compound IPSPs facilitate an intrinsic membrane potential oscillation in BLA principal neurons

**Figure 6:**
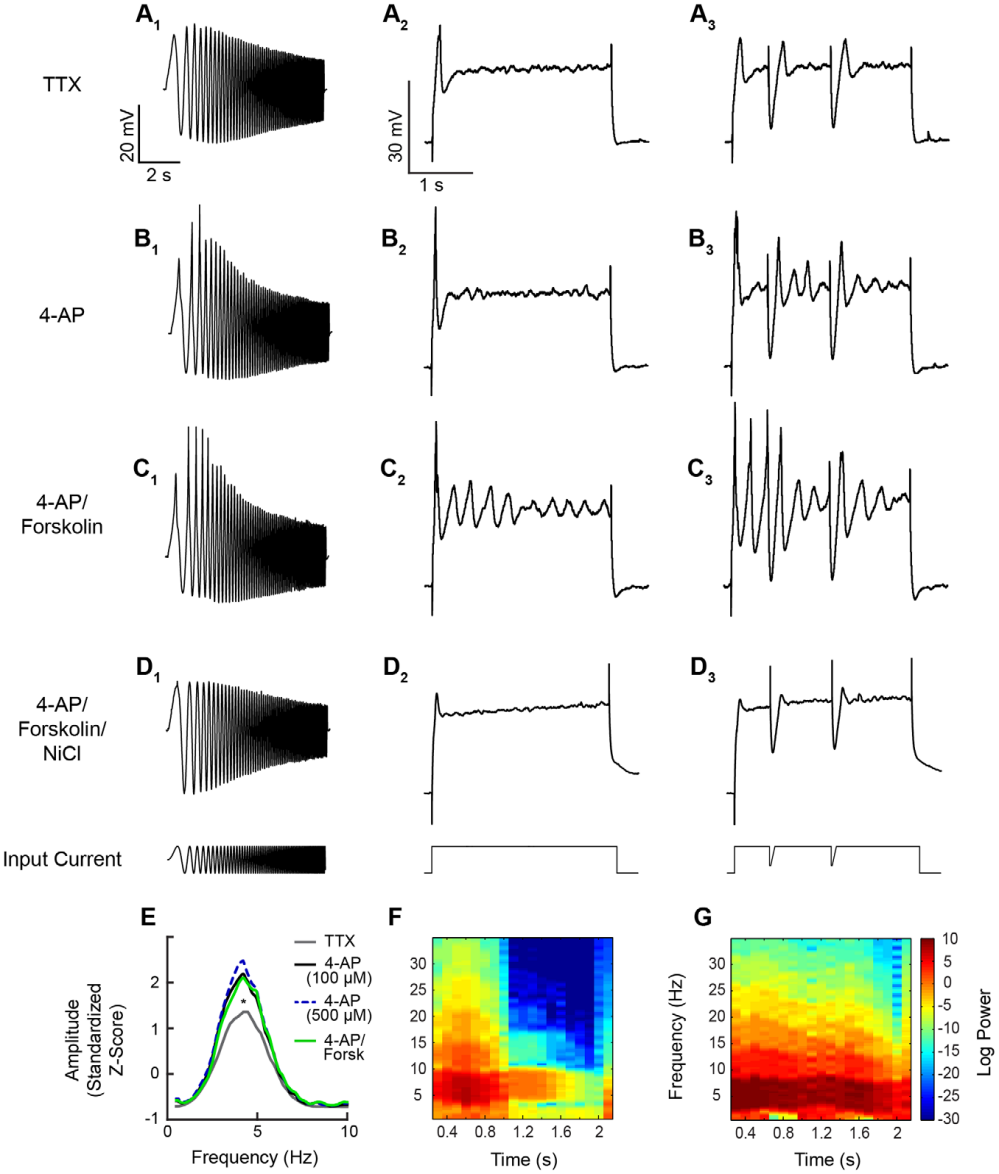
BLA principal neurons exhibited a modifiable intrinsic resonance and a membrane potential oscillation that was facilitated by compound IPSPs. (A1–D1) Principal neuron membrane potential response to injection of a sinusoidal current with constant amplitude and linearly changing frequency (0–12 Hz) in the presence of various drug cocktails. All neurons were held at baseline of −60 mV. (A1) Typical voltage response to the sinusoidal current in TTX (1 µM). The resonance of BLA principal neurons can be enhanced by application of 4-AP (B1, 500 µM) and the adenylyl cyclase activator, forskolin (C1, 10 µM), and is abolished by application of NiCl (500 µM, D1). Analysis of power spectra (E) shows that the enhancement of resonance by 4-AP and forskolin is significantly different from baseline (p<0.05). (A2–D3) Intrinsic membrane oscillations of BLA principal neurons, held at −60 mV, in response to a steady depolarizing current injection (A2–D2) and in response to the same current injection with superimposed IPSPs (A3–D3). Similar to resonant properties, membrane oscillations are enhanced by application of 4-AP and forskolin, and abolished in NiCl. Injection of artificial IPSPs in A3–D3 significantly enhanced the amplitude and duration of oscillations (F and G; spectrograms illustrate data from C2 and C3 respectively).

Most central nervous system neurons exhibit a preferred resonance frequency that provides them with the ability to filter synaptic input based on frequency [39]–[41]. Pape and colleagues have reported that principal neurons in the lateral amygdala of the cat have an intrinsic resonance frequency in the range of 1–3.5 Hz [33]. Here we extend these observations to show that BLA principal neurons of the rat also have an intrinsic resonance (**Figure 6A_1_–D_1_**, n = 8), with a preferred frequency at 4.2±0.1 Hz (**Figure 6E**).

Many of the membrane currents that contribute to the resonant properties of neurons have also been implicated in mediating long-lasting, sub-threshold MPOs in the BLA as well as other brain regions [32], [33], [39], [40], [42]. To determine whether compound IPSPs interact with an intrinsic MPO in principal neurons, we next examined the effect of IPSPs on membrane voltage in neurons depolarized to threshold in the presence of TTX (1 µM; n = 6). As illustrated in **Figure 6A_2_**, depolarizing current injection evoked a transient depolarizing voltage deflection at the onset of current injection but did not elicit an MPO in BLA principal neurons. Furthermore, injection of artificial IPSPs evoked a similar depolarizing voltage deflection on the rebound of each IPSP, but did not elicit an MPO (**Figure 6A_3_**). We hypothesized that the basal state of the neurons in the slice preparation might not be conducive to the expression of an MPO, and that modulation of intrinsic currents might be necessary to reveal the presence of an MPO.

#### The membrane potential oscillation is sensitive to modulation of its component currents

**Figure 7:**
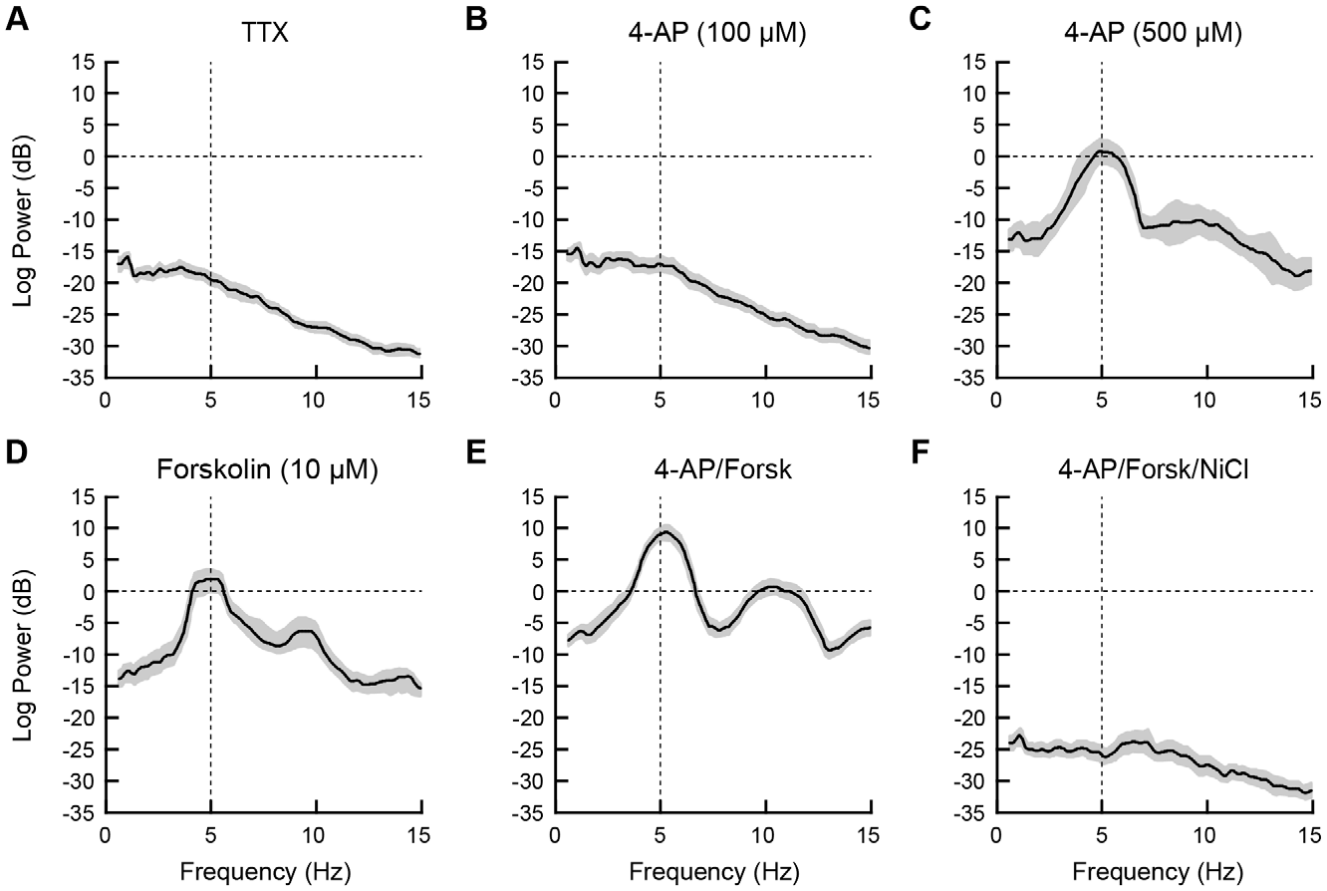
The peak power of the membrane potential oscillation was sensitive to modulation of I_A_ and I_T_ and activation of PKA. Power spectra of MPOs in BLA PNs in response to a depolarizing step with artificial IPSPs, with mean (solid lines) and 95% confidence intervals (shaded region). Frequencies at which the 95% confidence intervals do not overlap indicate statistically significant differences among the plots. (A) In the presence of TTX, neurons exhibit a weak MPO. (B,C) MPOs were not enhanced by bath application of 100 µM 4-AP (B) but were significantly enhanced by 500 µM 4-AP, with peak power at 4.9 Hz (C). (D) Application of forksolin, an activator of the c-AMP cascade, at 10 µM also enhanced a MPO with peak power at 4.8 Hz. (E) The MPO was significantly enhanced by a combination of 500 µM 4-AP and 10 µM forskolin, with peak power greater than for either drug alone but occurring at a similar frequency. (F) The MPO observed in forskolin and 4-AP was completely abolished by co-application of NiCl (500 µM) to block low-threshold calcium channels.

Work by Pape and colleagues has shown that MPOs in the BLA can be enhanced by modulating a select population of voltage-activated currents including, but not limited to, the hyperpolarization-activated cation current (I_H_) and the low-threshold Ca^2+^ current (I_T_) [43]. Significantly, an interaction between I_H_ and I_T_ is also thought to be a key element in the regulation of intrinsic resonance [39], [40], [42]. The I_T_ current is often opposed by the transient K^+^ current, I_A_ [44]–[47], which has been shown to regulate firing activity in BLA principal neurons [48]. Thus, we reasoned that blocking I_A_ channels could effectively enhance I_T_ and thus facilitate resonance behavior in BLA principal neurons and unmask an MPO. Bath application of the non-selective I_A_ channel blocker, 4-aminopyridine (4-AP), at 100 µM (**Figure 6E**) and 500 µM (**Figure 6B_1_ & 6E**) both significantly enhanced the amplitude of the peak resonance (One-way ANOVA, Tukey post-tests, F_3,46_ = 8.763, p<0.05). Application of 500 µM 4-AP also enhanced the expression of the transient depolarizing voltage deflection and unmasked a small, transient MPO at the onset of the depolarizing step (**Figure 6B_2_**). Furthermore, in the presence of 4-AP, the introduction of artificial IPSPs (**Figure 6B_3_**) enhanced the amplitude of the MPO, which had peak power at approximately 5 Hz (**Figure 7A–C**, n = 6).

Importantly, I_T_, I_H_, and I_A_ channels are all substrates for phosphorylation by protein kinase-A (PKA), which decreases the conductance of I_A_ channels and increases the conductance of I_H_ and I_T_ channels [49]–[55]. Thus, we next examined the effects of the PKA activator, forskolin, on the resonance properties of BLA principal neurons. As illustrated in **Figure 6C_1_**, bath application of forskolin (10 µM) in combination with 4-AP (500 µM) significantly increased the amplitude of the resonance peak compared to TTX controls (One-way ANOVA, Tukey post-test, F_3,46_ = 8.763, p<0.05). However, the peak power of the resonance in 4-AP and forskolin was not significantly different than that observed in the presence of 4-AP alone (**Figure 6E**). In the context of the depolarizing step, the addition of forskolin (10 µM) in combination with 4-AP (500 µM) enhanced both the amplitude and duration of the MPO in all neurons tested (**Figure 6C_2_**). The MPO resembled a damped oscillation [33] and, as can be seen in **Figure 6F**, the power of the MPO was greatest at the onset of the depolarizing current injection and declined over time. In the majority of neurons the MPO was seen to terminate before the conclusion of the depolarizing current injection. The introduction of artificial IPSPs further enhanced the oscillation (**Figure 6C_3_, G**) without changing the preferred frequency (**Figure 7E**, compared to **7C** and **7D**). Application of forskolin (10 µM) alone also unmasked an MPO, similar to the effects of 500 µM 4-AP, with a peak frequency at 4.8 Hz in all neurons tested (n = 4) (**Figure 7D**). Hence, activation of the cAMP-signaling cascade alone can facilitate the expression of the MPO in BLA principal neurons.

In other brain regions, MPOs are partially dependent on the activation of I_T_ channels, and as the transient depolarizing voltage deflections observed upon rebound from the IPSPs were reminiscent of low-threshold calcium spikes, we next determined whether blocking I_T_ channels with 500 µM NiCl [56] would inhibit the combined response to forskolin and 4-AP. Application of NiCl diminished the resonant properties of BLA principal neurons (**Figure 6D_1_**) and completely blocked the forskolin- and 4-AP-induced MPO in all neurons tested (n = 6) (**Figure 6D_2–3_** **,** **7F**), suggesting that an interaction between I_T_ and voltage-gated K^+^ channels, most likely I_A_ channels, play a critical role in MPO expression in BLA neurons.

**Figure 8:**
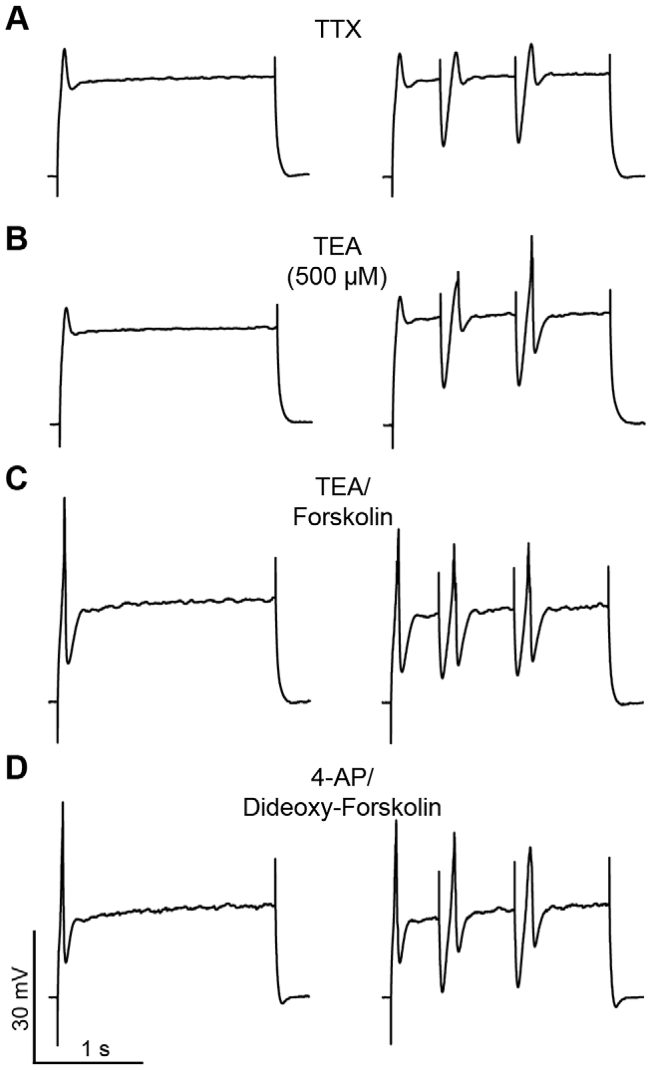
Forskolin and 4-AP modulation of the membrane potential oscillation were not mimicked by dideoxy-forskolin and TEA, respectively. Intrinsic membrane oscillations of BLA principal neurons, held at −60 mV, in response to a steady depolarizing current injection with and without artificial IPSPs. (A) Shows typical small membrane oscillations in TTX during the depolarizing current injection. In the presence of 1 µM TTX, the introduction of IPSPs evoked a transient depolarizing deflection at the termination of each IPSP, but failed to unmask a MPO. (B) MPOs are not enhanced by application of TEA (500 µM). (C) The addition of 10 µM forskolin had a small enhancing effect on MPOs in the presence of TEA. (D) Application of the inactive isomer dideoxy-forskolin in the presence of 4-AP did not enhance the MPO as observed previously with forskolin.

Application of high-micromolar 4-AP, however, can also block other K^+^ channels, including several that are also sensitive to micromolar concentrations of TEA. Hence, to determine if the effects of 4-AP on the MPOs resulted from a non-selective blockade of K^+^ channels, we repeated the experiments above in the presence of TEA (500 µM). As illustrated in **Figure 8**, application of TEA failed to mimic the 4-AP effect in either the presence or absence of simulated IPSPs. Moreover, concurrent application of forskolin (10 µM) and TEA also failed to unmask a significant increase in MPO amplitude over TEA alone (**Figure 8C**, n = 5), suggesting that the forskolin effect may only be observed when I_A_ channel activity is reduced by 4-AP.

To verify that the effects of forskolin were mediated by direct activation of the adenylyl cyclase-cAMP signaling cascade, we then examined the membrane response to application of the inactive forskolin isomer, dideoxy-forskolin (10 µM), in the presence of 4-AP. Dideoxy-forskolin failed to mimic the forskolin effect on MPOs in either the presence or absence of artificial IPSPs, suggesting that activation of the adenylyl cyclase-cAMP signaling cascade selectively facilitates IPSP-enhanced MPOs in principal neurons of the BLA (**Figure 8D**, n = 6).

**Figure 9:**
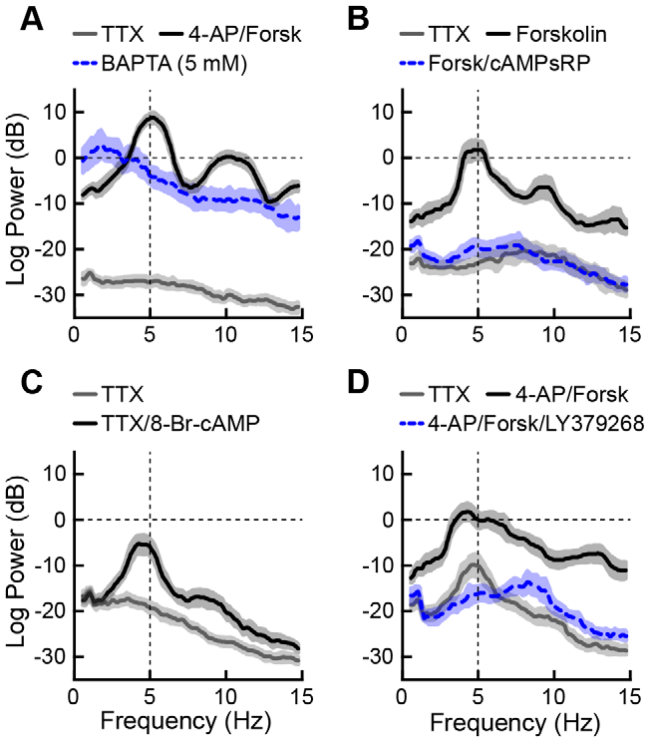
Membrane potential oscillations in the BLA were bi-directionally modulated by the adenylyl cyclase signaling cascade. Cumulative power spectra of intrinsic theta frequency MPOs in BLA principal neurons. Responses are plotted as mean (solid lines) and 95% confidence intervals (shaded regions). Frequencies at which the 95% confidence intervals do not overlap indicate statistically significant differences among the plots. (A) BAPTA-containing patch solutions disorganized the frequency tuning of 4-AP- and forskolin-induced MPOs. (B) Inhibiting PKA activation completely abolishes forskolin-induced MPOs. (C) Activation of PKA with the cAMP analog 8Br-cAMP induces MPOs in TTX alone that are similar to those observed in response to forskolin. (D) Activation of mGluR II glutamate receptors with LY379268 completely blocked 4-AP and forskolin-induced theta MPOs.

Finally, we examined if modulation of intracellular Ca^2+^ levels also play a role in regulating the MPO. Here, inclusion of the Ca^2+^ chelator, BAPTA (5 mM), in the patch solution completely blocked the MPO induced by co-application of 4-AP (500 µM) and forskolin (10 µM) (**Figure 9A**, n = 6), suggesting that fluctuations in intracellular Ca^2+^ levels also play an important role in the expression of MPOs in BLA principal neurons. However, this result raised the possibility that the drug-induced MPO may be independent of activation of the cAMP-PKA signaling cascade. To address this question, we included the competitive antagonist of cAMP-induced PKA activation, cAMPs-RP, in the patch solution. Inclusion of cAMPs-RP (25 µM) completely blocked the MPO induced by forskolin (**Figure 9B**, n = 4). Conversely, inclusion of a non-hydrolysable cAMP analogue, 8-Br-cAMP (5–10 µM), in the patch pipette unmasked an MPO in the presence of TTX alone that was similar in magnitude to that induced by forskolin (**Figure 9C**, n = 6). Hence, Ca^2+^ influx through I_T_ channels, elevation of intracellular Ca^2+^, and activation of the adenylyl cyclase-cAMP-PKA signaling cascade each play an important role in the expression of MPOs in BLA principal neurons.

The sensitivity of the MPO to modulation by intracellular Ca^2+^ and activation of the cAMP-PKA signaling cascade suggested that receptors coupled to G_αs_ would facilitate MPOs, whereas those coupled to G_αi_ would attenuate MPOs. To test this hypothesis, we examined the effect of prior application of the selective mGluR2/3 agonist, LY379268, on the 4-AP- and forskolin-induced MPOs. Principal neurons of the BLA express high levels of mGluR2/3 receptors [57], [58], which couple to G_i/o_ proteins to inhibit adenylyl cyclase activity [59], and we reasoned that activation of these receptors would attenuate drug-induced MPOs. As illustrated in **Figure 9D** (n = 10), application of LY379268 (50 µM) completely blocked the MPOs.

## Discussion

In the present study, we demonstrate that spontaneous, compound IPSPs function to increase spike-timing precision both within and across BLA principal neurons. Previous studies have shown that these IPSPs are driven by local, burst-firing PV^+^ neurons [18], which have a high level of connectivity with BLA principal neurons. These data suggest that spontaneous, compound IPSPs would function to synchronize action potentials in a large population of principal neurons. We also show that compound IPSPs promote and entrain a high delta/low theta frequency membrane potential oscillation (MPO) that is uncovered by activation of the cAMP-PKA signaling cascade. The oscillatory nature of BLA principal neurons is also manifested as a modifiable inherent resonance frequency. We propose that the interaction of compound IPSPs with the oscillatory properties of BLA principal neurons is a viable mechanism for synchronizing firing activity in this cell population, promoting network oscillations within the BLA, and enhancing coherent oscillations between the BLA and other brain regions involved in fear.

### Synchronized inhibition drives coordinated activity of BLA principal neurons

Recent evidence suggests a wide variety of behaviors require synchronized neural activity and network oscillations, both of which are promoted by synaptic inhibition [13]–[17]. Here, we demonstrate that BLA principal neurons receive highly synchronized, rhythmic inhibition which, in turn, synchronizes firing activity among groups of BLA principal neurons. Importantly, spontaneous activity of interneurons in the prefrontal cortex at theta frequency entrains the firing of principal neurons to an ongoing network theta oscillation [60]. This example from the prefrontal cortex suggests the coordination of principal neuron firing by inhibition is critical for salient output of some neural circuits. Through coordinating the firing of large groups of BLA principal neurons, compound IPSPs should improve salience by promoting summation of output and leading to spike-timing dependent plasticity in both the BLA and its targets.

In order to study the effect of compound IPSPs on spike-timing precision, we used two proxies: artificial IPSPs generated by direct current injection at the soma, and compound IPSPs evoked by direct stimulation of interneurons in the BLA under glutamatergic blockade. We showed that spike-timing precision within single neurons is improved by spontaneous IPSPs, artificial IPSPs, and stimulation-evoked IPSPs, with artificial IPSPs being significantly more effective than evoked IPSPs. Furthermore, artificial IPSPs were able to significantly coordinate firing across neurons, but evoked IPSPs were not, due to the observed variability in the waveform. Spontaneous, compound IPSPs observed across pairs had a highly consistent waveform (evident in a representative pair in **Figure 1A**), likely because they are generated by burst-firing PV^+^ interneurons, which innervate BLA principal neurons perisomatically and have their activity coordinated through a syncytium. In contrast, stimulation of the BLA to evoke IPSPs probably recruited multiple subtypes of GABAergic interneurons targeting multiple compartments of the principal neurons [61]–[63] and hence introduced variability across cells in the IPSP waveform. While PV^+^ interneurons seem uniquely positioned to generate synaptic inhibition that is ideal for interacting with an MPO and coordinating activity of BLA principal neurons, the possibility is not excluded that other inhibitory input, for instance feed-forward inhibition from cortical or thalamic sources [64], [65], could exert a similar coordinating influence.

The fact that artificial IPSPs were able to mimic the effects of evoked and spontaneous IPSPs on spike-timing precision without directly influencing the membrane conductance suggests they act primarily via membrane hyperpolarization. This hyperpolarization likely causes activation of I_H_ and de-inactivation of voltage-gated currents including I_T_, which would contribute to calcium spikes upon rebound [42]. Because I_T_ is typically inactive near resting membrane potential, the observed effect of compound IPSPs on spike timing is probably more applicable when BLA principal neurons are depolarized from rest. It is also important to consider that compound IPSPs occur amidst ongoing synaptic activity, not in the absence of synaptic input as when tested here. In the *in vivo* system, compound IPSPs may not produce spikes in the absence of excitatory transmission, but rather interact with ongoing synaptic activity to influence the timing of spikes.

The ability of compound IPSPs to coordinate spiking activity most likely occurs across large groups of BLA principal neurons due to the broad connectivity of PV^+^ interneurons [38], the synchronization of PV^+^ interneuron firing activity through a syncytium [23]–[25], and, as shown here, the robustness of IPSP coordination of spike timing across principal neurons despite varying intrinsic properties. Although synchronizing large networks of principal neurons will improve potency of efferent signaling, it could also limit the specificity of signaling. Cortical inputs to the BLA are organized topographically [66], and synchrony throughout the nucleus could weaken the specificity afforded by this topography. A loss of specificity in this circuit through excessive synchronization within the amygdala may lead to generalization of fear learning, which has been implicated in affective disorders such as post-traumatic stress disorder [67]. Furthermore, less than a quarter of BLA neurons appear to be incorporated into the engram for any specific fear memory [68], [69]. If encoding and recall of fear memories depend on network oscillations, there must be a mechanism to preferentially incorporate some neurons while excluding others. Some potential mechanisms include regulation of the extent of the syncytium or of projections from the PV^+^ interneurons onto principal neurons, or, more interestingly, interactions between variability in the frequency of the network oscillation with variations in preferred resonance frequency of the principal cells.

Considering the prominent role inhibition appears to play in coordinating the activity of BLA principal neurons, it is likely that stimuli altering the frequency of IPSPs *in vivo* could drastically change the output activity of the BLA. For instance, activation of serotonin 2A or cholecystokinin B receptors, both of which are implicated in emotional learning [70], increase the frequency of rhythmic IPSPs in BLA principal neurons through indirect excitation of interneurons [18], . A similar effect is observed in the BLA in response to local release of dopamine in mice [20] and primates [19]. Moreover, the BLA receives dopaminergic input from the ventral tegmental area, which also exhibits a network oscillation at 2–5 Hz during working memory tasks [72], raising important questions about the nature of the interaction of phasic dopamine release with a BLA circuit that itself generates rhythmic activity.

### Resonance frequency and intrinsic membrane oscillations in BLA principal neurons

In the present study we have shown that BLA principal neurons in the rat have an intrinsic resonance that was extremely consistent, with nearly all neurons displaying a peak resonance between 4.2 and 4.4 Hz. This intrinsic resonance was insensitive to application of TTX (1 µM), whereas a previous study in guinea pigs reported neurons in the lateral and basolateral nuclei of the amygdala express a TTX-sensitive inherent resonance frequency at 2.5 Hz [33]. The difference in reported resonance frequencies is likely due to the different model species, as we have also seen differences in peak resonance frequency of principal neurons between rat and primate (unpublished observation). The difference in TTX sensitivity, however, is likely explained by the concentrations of TTX employed. In the study by Pape and colleagues the resonance frequency was abolished by 20 µM TTX, compared to the 1 µM TTX used here. High concentrations of TTX are known to block the persistent Na^+^ current, and future studies should investigate whether it contributes to resonance in BLA principal neurons, as it does in LA neurons [32]. Similar to our observations, hippocampal principal neurons also display resonance that is insensitive to 1 µM TTX with a peak at 4.1 Hz [73].

In addition to selectively filtering synaptic input in high delta/low theta bands, BLA principal neurons also express high- and low-threshold MPOs in this frequency range, as described by Pape and colleagues [32]. Here we show the presence of an MPO that occurs at the peak resonance frequency of these neurons (∼4–5 Hz) and seems to share some mechanisms with both previously described oscillations. Although Pape and colleagues found no effect of specific Ca^2+^ channel blockers on the high threshold membrane oscillations [33], recordings with a BAPTA-containing electrode completely abolished the oscillation. In our hands, bath application of NiCl completely abolished the MPO, suggesting a strong influence of T-type Ca^2+^ channels. The Pape study also reported that high-threshold membrane oscillations were insensitive to 10 mM 4-AP, suggesting that voltage-gated K^+^ channels were not involved in that membrane oscillation [33]. We observed, however, that application of 100–500 µM 4-AP significantly enhanced the membrane oscillations, suggesting I_A_ may actively suppress the MPO, acting in opposition to I_T_. This could also be related to changes in input resistance, but the lack of effect of 500 µM TEA suggests a specific role of I_A_. A similar relationship between I_T_ and I_A_ has been shown in other systems [47], [74], and factors that either enhance I_T_ or reduce I_A_ could then unmask the expression of the intrinsic membrane oscillations. In agreement with Pape and colleagues, we did not find an effect on intrinsic membrane oscillations of blocking I_H_ with ZD7228 (60 µM, data not shown). While this is not an exhaustive pharmacological characterization, we believe we have identified the major currents involved in mediating this MPO. Other currents, including the persistent sodium current and calcium-activated potassium currents may also be involved [33], and future study to illuminate their roles in this phenomenon would be valuable.

It is notable that the currents mentioned above (I_T_, I_A_, and I_H_) are all sensitive to membrane hyperpolarization, particularly in the voltage range between rest and action potential threshold [75]–[77]. Specifically, I_T_ channels are de-inactivated by hyperpolarization in this range and I_H_ channels are activated, while I_A_ channels are activated by depolarization in this range. We have shown that compound IPSPs facilitate the MPO in the absence of spiking, and this is likely due to hyperpolarization-mediated de-inactivation of I_T_ and activation of I_H_. While we did not find an effect of I_H_ blockade on the MPO, it is possible this is an artifact of the degree to which we depolarize the membrane to enhance the MPO. The MPO is likely also active in a more subtle form at membrane potentials only slightly depolarized from rest, where I_T_ and I_A_ are active and I_H_ would enhance the rebound from an IPSP and may contribute directly to the MPO.

In addition, the conductances of these currents, and therefore the magnitude of the MPO itself, are not fixed but sensitive to modulation. Importantly, the channels mediating I_T_ and I_H_ increase their activity in response to PKA phosphorylation [49]–[54]. Conversely, activity of K^+^ channels mediating I_A_ is decreased by PKA phosphorylation [55]. Together, these would enable neurotransmitter systems which modulate PKA activity to have synergistic effects to bi-directionally modulate the MPO.

The frequency of rhythmic, compound IPSPs is also sensitive to modulation. We have previously shown that dopamine acts to increase IPSP frequency into a range of 2–6 Hz [18]–[20]. This would bring the IPSP frequency closer to the peak resonance frequency of BLA principal neurons, ensuring principal neurons respond to incoming rhythmic IPSPs with maximal voltage deflections and also serve to enhance the interaction with the MPO. In our hands, the oscillation does not occur spontaneously but is initiated by the IPSP and is naturally damped, quickly decaying from its initial amplitude. More frequent synchronized IPSPs would better reset drift in the phase relationship between cells and better maintain the amplitude of the oscillation.

It is interesting to note that the intracellular cascades activated by many of the neuromodulators that promote the MPO (e.g., G_s_-coupled activation of cAMP and PKA) are also critical for synaptic plasticity. Through this mechanism, cells primed by neuromodulators to exhibit an MPO may be more likely to contribute to a fear memory engram.

#### Implications for learning and memory

Recent studies by several groups have emphasized the importance of amygdala network oscillations and synchronized oscillations across multiple brain regions in regulating long-term fear memory [43], [78]–[84]. Importantly, phase-locked stimulation of the amygdala and hippocampus at theta frequency during extinction training prolongs fear expression, suggesting synchronized network oscillations between these regions are an essential neurological component of fear memory [9]. The high delta/low theta oscillations in the LFPs of the BLA, hippocampus, and prefrontal cortex during fear acquisition and expression [7], [8] match the frequency of the MPO and the peak resonance in BLA neurons, suggesting the intrinsic properties of BLA neurons contribute to the network oscillation. As we have argued, a candidate mechanism to promote these network oscillations is the interaction of synchronized IPSPs with MPOs in BLA principal neurons. MPOs could contribute to fear learning by promoting network oscillations, and by improving spike-timing precision they could support fear memory formation through enhanced spike-timing dependent plasticity [29], [85].

The sub-cellular mechanism of the intrinsic MPO is well-suited to facilitate plasticity in the BLA and thereby promote fear learning. The MPO requires activation of voltage-gated calcium currents [33], causing calcium influx and subsequent activation of cAMP and PKA [86]. This can, in turn, reinforce the oscillation through phosphorylation of ion channels. In fact, the oscillation is weak or nonexistent under our baseline experimental conditions, but must be uncovered by application of the PKA activator, forksolin. The close relationship of the MPO with the adenylyl cyclase-cAMP signaling cascade is particularly important because its downstream targets have been implicated in fear learning and memory [87]–[90], and in regulating theta oscillations in the amygdala *in vivo* [43], [87]–[89], [91]. It is also noteworthy that downstream targets of this signaling cascade, particularly the cAMP response element binding protein (CREB), have been used to identify those neurons activated specifically during fear memory formation [68], [69].

One neurotransmitter receptor known to modulate the cAMP-PKA pathway, the dopamine D1 receptor, is also implicated in fear learning. Release of dopamine and subsequent activation of D1 receptors in the BLA are critically involved in the acquisition and consolidation of fear memory [92], [93]. Additionally, we have recently shown that D1-receptor activation is necessary for long-term potentiation of sensory afferents to the BLA [94]. Aside from direct effects on synaptic plasticity, D1 receptor activation may promote fear learning by facilitating an MPO. Considering that the MPO must be uncovered by activation of PKA *in vitro*, D1-receptor activation could provide the requisite PKA activation to initiate a self-reinforcing high delta/low theta oscillation *in vivo*. Importantly, in the prefrontal cortex, application of dopamine mimics the effect of a working memory task to entrain firing of principal neurons to an ongoing theta oscillation [60]. One possible explanation, which parallels our observations in the BLA *in vitro*, is that interneurons maintain a network oscillation by providing a background of rhythmic activity to which principal neurons are, at baseline, minimally sensitive. In this model, the principal neurons become more sensitive to rhythmic inhibitory input from the interneurons by activation of PKA, either directly (as in our hands, with forskolin) or with the introduction of dopamine (either artificially or endogenously through a behavioral task) [60], [95]. Interestingly, activation of D1 receptors has also been shown to enhance spike-timing dependent plasticity, potentially compounding with the effects of D1 activation on spike-timing precision via the MPO [96].

We have shown that inhibition of adenylyl cyclase and cAMP production by activation of group II metabotropic glutamate receptors completely abolished the high amplitude MPO induced by forskolin and 4-AP. Activation of these receptors has been associated with reductions in fear learning, as well as de-potentation of synapses and long-term depression [59], [97], [98], providing further support for a role for MPOs in BLA-dependent fear learning. Interestingly, the G_i_-coupled type 1 cannabinoid receptor has been shown to reduce neural synchrony and dampen theta and gamma oscillations in the hippocampus [99], further suggesting changes in cAMP levels can bi-directionally modulate the propensity of a network to oscillate.

While network oscillations contribute to normal brain functions, including fear learning, aberrant oscillations have been implicated in the pathophysiology of psychiatric disorders. For example, it is well established that diminished synchrony between pyramidal neurons, and consequently aberrant network oscillations in the gamma band, are involved in the pathophysiology of schizophrenia [100]. Interestingly, the changes in oscillations observed in schizophrenia have been specifically linked to diminished function in the PV^+^ subpopulation of interneurons in the cortex [100]. It is worth noting that theta oscillations are thought to modulate the gain of gamma oscillations, and both are produced through the action of PV^+^ interneurons [101]–[103]. Gamma-frequency oscillations are observed in the BLA both *in vivo* and *in vitro* [104], [105], and may be generated by similar mechanisms in the BLA as in the cortex due to their similar composition and architecture [106]. Considering the importance of neural oscillations and that compound IPSPs may influence gamma oscillations through their effects on high delta/low theta oscillations, future studies should address changes in oscillations and PV^+^ interneurons in the amygdala in various psychiatric disorders, particularly post-traumatic stress disorder and others linked to fear learning [67].
